# Hostile media perception affects news bias, but not news sharing intentions

**DOI:** 10.1098/rsos.211504

**Published:** 2022-04-20

**Authors:** Sergio Lo Iacono, Terence Daniel Dores Cruz

**Affiliations:** ^1^ Umeå University, Umeå, Sweden; ^2^ University of Essex, Colchester, UK; ^3^ Organization Sciences, Vrije Universiteit Amsterdam, Amsterdam, Noord-Holland, Netherlands

**Keywords:** hostile media perception, news bias, news sharing, trust

## Abstract

Hostile media perception (HMP) theory suggests that partisans perceive neutral coverage of news by outlets opposite to their political leaning as biased against their side. We conducted two pre-registered online experiments to assess the effect of HMP on news bias and news sharing intentions regarding two salient and controversial topics in the US: police conduct (Study 1, *N* = 817) and COVID-19 norms (Study 2, *N* = 819). Results show that partisans perceive neutral coverage of news by outlets opposite to their political leaning as biased, even when we account for their prior beliefs regarding the media outlet and news content. However, HMP seems to be limited in its consequences, as it has little impact on partisans' willingness to share news from outlets of opposite political leaning, even though the news is perceived as biased.

## 1. Theoretical background

Trust in the media has been declining in the USA over the last three decades as news outlets are seen as increasingly untrustworthy by the general public [1]. Evidence shows that this drop in trust is closely linked to partisanship and polarization of the political and media discourse [2]. Fox News, for instance, is viewed at the same time as the most and least trusted source of news depending on respondents' political orientation [3]. Yet, mainstream media are generally evaluated as more trustworthy than hyper-partisan outlets, covering an essential role in the public discourse [4].

Hostile media perception (HMP) theory posits that partisans perceive identical neutral news coverage from sources seen as opposite to their political leaning as biased across a variety of controversial topics. HMP effects have been empirically identified as regards security, elections, sports and so on [5–10]. However, some of the most recent and pressing issues in the US society have not received much scrutiny in this regard yet.

Over the past years, the deadly use of force from the US police has attracted worldwide media attention, sparking fierce discussions and intense public examination [11–13]. The debate has developed along party divides, affecting public sentiments: while Democrats' confidence in the police has declined over the years, Republicans' confidence has remained rather stable [14–16]. Similarly, with the outbreak of the COVID-19 pandemic, the increasing death toll and the harm on the economy [17], Republican and Democrat representatives in the US House and Senate have quickly polarized, failing to reach political consensus on the matter [18]. As both debates have rapidly escalated along party lines, the news coverage from mainstream media is likely to trigger a HMP effect whereby partisans will perceive neutral news coverage as biased, potentially further harming trust towards principal information providers.

In this study, we assess whether there is a HMP effect with respect to two recent and controversial topics in US society, namely police conduct and COVID-19 norms. To do so, in line with previous research on HMP, we randomly assign subjects to identical neutral news coverage from two mainstream media, i.e. Fox News and CNN, and analyse how people from opposite political leanings react to it.

As pointed out by recent contributions in politically motivated cognition, such randomized experiments can violate the ‘excludability assumption' since the assignment to treatment may alter other factors (alongside political motivation) that influence the outcomes, making the design confounded [19]. That is, the effect of the treatment on the outcomes might not be exclusively due to respondents' political leaning (which can be hardly randomized). For instance, being exposed to information that is incoherent with prior opinions on the media outlet may affect the respondent's receptivity to the treatment and his/her consequent evaluation of the news. Similarly, prior feelings on the specific issue at hand could affect one's positive or negative reaction to the information received [19,20]. As such prior beliefs and attitudes towards news topic (e.g. attitudes on police conduct or COVID-19 norms) and news source (e.g. Fox News and CNN) may correlate with both the respondents' political leaning and news bias and sharing intentions, not accounting for such variables could compromise our ability to properly identify the causal relationship. In this regard, we improve on previous experimental research on HMP and, following Arpan & Raney [10] and Peterson & Kagalwala [21], we account for prior beliefs concerning media bias and news content by including relevant statistical controls (see Material and methods for more details). Thus, building upon past research on news bias and politically motivated cognition, we argue that if HMP correctly identifies a causal effect of news source *and* political leaning on news bias [19,20,22,23], we should observe the following:

[H1] Partisans exposed to the same neutral coverage of news from a source opposite to their political leaning will report the news as more biased, even when accounting for subjects' prior beliefs regarding the media outlet and the content of the news.

If political stance does lead people to evaluate news on the basis of the source rather than its content, then we can expect HMP to have broader attitudinal and behavioural consequences [8,9]. In particular, neutral coverage of news by outlets opposite to people's political leaning could also affect one's willingness to spread such information [19,20]: reading news from a source perceived as politically biased might lead us to ignore it entirely, decreasing our willingness to share it with others. Assuming the same news content, we should observe people to express a stronger inclination to spread news from sources they agree with from a political point of view. As above, such an effect should be independent from prior beliefs on the media outlet or news content to be causally valid. That is, we hypothesize that

[H2] Partisans exposed to the same neutral coverage of news from a source opposite to their political leaning will be less willing to share the news, even when accounting for subjects' prior beliefs regarding the media outlet and the content of the news.

## 2. Overview of studies

To test our hypotheses, we conducted two pre-registered experiments on Amazon Mechanical Turk (AMT), assessing the consequences of HMP on news bias and intentions to share news for two topics: police conduct (Study 1, *N* = 817) and COVID-19 norms (Study 2, *N* = 819). Each study has a 2 × 2 between-subjects design where we manipulated news source and news content, randomly assigning participants to each condition. Subjects were asked to evaluate news bias across eight different dimensions in accordance with prior literature [24]. We manipulated the news content to evaluate the robustness of the HMP effect across situations that differ in valence. The news content chosen for the treatments was always real and covered by both CNN and Fox News. Subjects participated in a screening survey gathering relevant information on respondents' political leanings, demographics and prior beliefs about media outlets, police and COVID-19, two to three weeks before the experiment.

Our hypotheses, experimental design and analysis plan were pre-registered on Open Science Framework, where we have also deposited materials, electronic supplementary material with descriptive statistics, additional analyses and robustness checks.

## 3. Study 1: police conduct

### 3.1. Material and methods

#### 3.1.1. Experimental design

Study 1 had the following structure. First, subjects participated in a screening survey where we gathered a variety of information on respondents' political orientation, beliefs on media bias, trust, attitudes concerning the legal system, and demographics. After two to three weeks, participants were invited to the experiment where they were randomly assigned to a treatment. In the experiment, subjects first read the news from a mainstream outlet, and then filled out a questionnaire. The study had a 2 × 2 between-subjects design where we manipulated news source (Fox News versus CNN) and news content (police positive versus negative conduct), table 1.

**Table 1.** Between-subjects design of Study 1.

		news source	
		Fox News	CNN
news content	police officer killing a civilian	Fox News, police officer killing a civilian	CNN, police officer killing a civilian
	police officer saving the life of a civilian	Fox News, police officer saving the life of a civilian	CNN, police officer saving the life of a civilian

**Figure 1.**
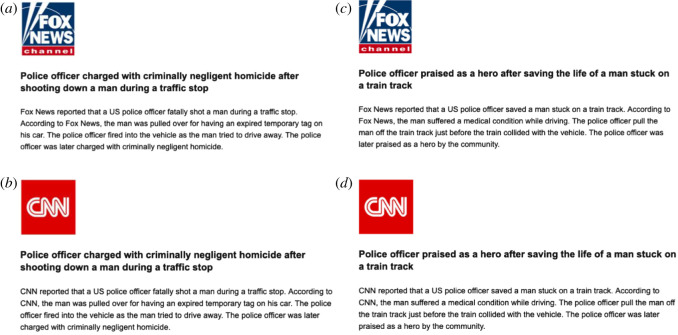
Treatments in Study 1 ‘Police conduct'. (*a*) Fox News, negative police conduct; (*b*) CNN, negative police conduct; (*c*) Fox News, positive police conduct; (*d*) CNN, positive police conduct.

The news showed to participants was a short report with a brief headline (about 600 characters max)—figure 1. The news coverage was always neutral—i.e. it conveyed an event that was positive or negative in content, but the event was not reported in a biased manner for any particular side. Both news items presented were real, and we provided the link to the original article upon request to all interested participants. After the treatment, subjects completed a battery of questions, including their willingness to share the news, their perception of media bias, trustworthiness of particular news and so on, using the same measures of the screening survey (where relevant).

#### 3.1.2. Participants and procedures

Data were collected between 16 June and 12 July 2020. The questionnaire was prepared in Qualtrics and ran on AMT [25–29]. We paid participants US federal minimum wage. To help ensure data quality, we recruited MTurkers who had a certified and long-lasting history of consistent work (i.e. more than 98% of previous assignments approved and participation in at least 500 past assignments) in line with prior research [25,30,31]. We also restricted our sample to participants from the US [32]. In addition to pre-screening strategies, we prevented re-takings by registering MTurkers' Worker ID after each session and blocking participants who had already participated in the study. Note that only MTurkers who took part in the screening survey could participate in the experiment. The sample size was determined on the basis of previous studies and standards in the field [6,8,9,33,34].

The screening survey was completed by 997 participants who were then invited to the experiment; 822 MTurkers returned for the experiment (participation rate = 82.4%). Participants who did not answer questions measuring our dependent variables (DVs) were excluded from the analyses, giving us a total sample size of 817 subjects for news bias and 816 subjects for news sharing intentions. Table 2 provides descriptive statistics of our sample.

**Table 2.** Study 1 ‘Police Conduct' Descriptive statistics. CNN and Fox bias, as well as political leaning (i.e. conservative scale), police attitudes, US division and demographics were measured in the screening survey.

variables	*M*	*s.d.*	*min*	*max*	*N*
news bias	1.48	0.85	0	4	817
news sharing intentions on social media	1.34	0.95	0	3	816
conservative scale	3.65	1.86	1	7	817
education					
high school diploma or less	0.07	0.26	0	1	817
some college	0.16	0.36	0	1	817
associate degree (2 years) or spec. technical training	0.11	0.32	0	1	817
bachelor's degree	0.42	0.49	0	1	817
some graduate training	0.03	0.17	0	1	817
graduate/professional degree or higher	0.21	0.41	0	1	817
ethnicity					
white	0.73	0.44	0	1	817
black	0.13	0.34	0	1	817
hispanic or latino/latina	0.04	0.20	0	1	817
asian	0.08	0.27	0	1	817
other	0.02	0.13	0	1	817
gender					
male	0.49	0.5	0	1	817
female	0.51	0.5	0	1	817
other	0.00	0.08	0	1	817
age	42.29	12.88	19	77	817
police is doing a good job	3.21	1.23	1	5	817
trust towards police	5.23	3.13	0	10	817
Fox bias	2.66	1.25	0	4	817
CNN bias	2.02	1.19	0	4	817
US Division					
New England	0.05	0.21	0	1	817
Mid-Atlantic	0.13	0.34	0	1	817
East North Central	0.14	0.35	0	1	817
West North Central	0.06	0.24	0	1	817
South Atlantic	0.24	0.43	0	1	817
East South Central	0.05	0.23	0	1	817
West South Central	0.11	0.31	0	1	817
Mountain	0.06	0.25	0	1	817
Pacific	0.13	0.34	0	1	817
Puerto Rico and other US territories	0.02	0.15	0	1	817

#### 3.1.3. Measures

*News bias.* News bias was measured using an index computed from the standard eight items scale (0 = news is unbiased, 4 = news is biased) as developed by Yale *et al*. [24]. The scale asked respondents to think about the coverage of the story they just read and indicate whether each of the following words described their feelings on a 5-point Likert scale (1 = strongly disagree, 5 = strongly agree): [Do you think the coverage of this story is…] Balanced, Reporting the whole story, Objective, Fair, Accurate, Honest, Believable, Trustworthy (news bias, *α* = 0.94). We followed the same approach to measure prior bias towards Fox News and CNN in the screening survey. The only difference was that respondents were asked to think about the given media outlet as a source of news and information instead of thinking about the specific news (Fox News bias *α* = 0.98; CNN bias *α* = 0.98).

*News sharing intentions.* News sharing intentions on social media were measured using the question ‘How willing are you to share this news on social media?'. The question was answered on a 4-point scale (0 = very unwilling, 3 = very willing) and relies on the work of Mosleh *et al*. [35]. Asking sharing intentions in such a way has been shown to approximate actual sharing activity on social media [35]. News sharing intentions in conversations was measured following the same approach (replacing ‘on social media' with ‘in conversations').

*Political leaning and party identity.* Political leaning was measured using the conservative scale: ‘Below you can see a seven-point scale on which the political views that people might hold are arranged from 1 = extremely liberal to 7 = extremely conservative. Where would you place yourself on this scale?'. The conservative scale was centred around the mean in the regression models. We also measured party identity with the question ‘Generally speaking, do you usually think of yourself as a Republican, Democrat, Independent, or something else?'. Both questions are taken from the General Social Survey.

*Police attitudes.* We employed two measures, adapted from the General Social Survey, to control for prior attitudes towards police conduct: ‘Police are doing a good job' and ‘Trust in the police'. ‘Police are doing a good job' was measured using a 5-point scale (1 = very bad job; 5 = very good job) and it was based on the following question ‘Taking into account all the things the police are expected to do, would you say they are doing a good job or a bad job?’. ‘Trust in the police' was measured on an 11-point scale (0 = no trust at all, 10 = complete trust) and it relied on this question ‘Please indicate on a score of 0–10 how much you personally trust each of the following institutions… the police'.

## 4. Results and discussion

To test the HMP argument, we estimated the effect of the interaction between political leaning and treatment condition on news bias and news sharing intentions via OLS regression. More specifically, we computed the difference between the slopes of political leaning across treatment conditions with the same content (e.g. positive police conduct) but different news source (i.e. Fox News versus CNN)—i.e. the second difference [36]; see tables 3 and 4.

**Table 3.** Study 1 ‘Police conduct'—Effect of conservative leaning on news bias within and between treatments. Linear effects within and between treatments are computed using average marginal effects in Stata 16.1 employing the *margins* and *mlincom* commands. Robust standard errors in parentheses.

DV: news bias	linear effect within treatments (first difference)		linear effect between treatments (second difference)
conservative in Fox police neg	0.022 (0.032)	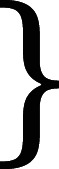	0.146*** (0.044)
conservative in CNN police neg	0.168*** (0.036)		
conservative in Fox police pos	−0.105** (0.035)	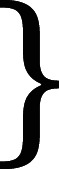	0.054 (0.041)
conservative in CNN police pos	−0.051 (0.033)		

*p ≤ 0.05, **p ≤ 0.01, ***p ≤ 0.001 (for two-sided tests).

**Table 4.** Study 1 ‘Police conduct'—Effect of conservative leaning on news sharing intentions on social media within and between treatments. Linear effects within and between treatments are computed using average marginal effects in Stata 16.1 employing the *margins* and *mlincom* commands. Robust standard errors in parentheses.

DV: news sharing	linear effect within treatments (first difference)		linear effect between treatments (second difference)
conservative in Fox police neg	−0.101* (0.040)	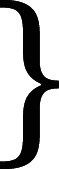	−0.038 (0.047)
conservative in CNN police neg	−0.139*** (0.037)		
conservative in Fox police pos	0.058 (0.041)	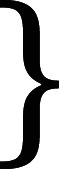	−0.012 (0.050)
conservative in CNN police pos	0.046 (0.039)		

*p ≤ 0.05, **p ≤ 0.01, ***p ≤ 0.001 (for two-sided tests).

**Figure 2.**
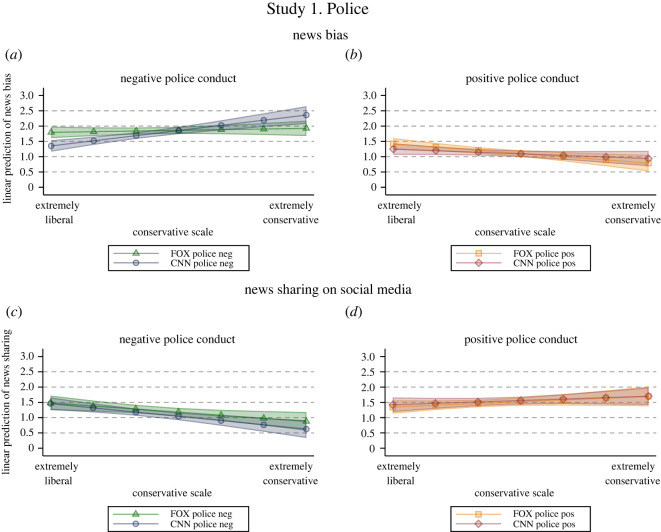
Study 1 ‘Police conduct'—Linear prediction of news bias and news sharing intentions by political leaning and treatment. Linear prediction of perceived bias of news coverage (*N* = 817, *R^2^* = 0.33; (*a*,*b*)) and news sharing intentions on social media (*N* = 816, *R^2^* = 0.27; (*c*,*d*)) by political leaning and treatment with 95% CIs based on OLS regression with robust standard errors and controls for demographics, US state, prior media bias and attitudes towards the news content.

Figure 2 shows the linear prediction of perceived bias of news coverage (*N* = 817, *R^2^* = 0.33; (*a*,*b*)) and news sharing intentions on social media (*N* = 816, *R^2^* = 0.27; (*c*,*d*)) by treatment and political leaning, estimated via OLS regression with controls for prior media bias and attitudes towards the police, as well as US State and demographics. Complete regression results and robustness checks are available in the electronic supplementary material.

Our findings indicate that people with stronger conservative leaning exposed to neutral coverage of negative police conduct (i.e. police officer kills civilian) from CNN perceived the story as more biased than subjects with more liberal views (*β* = 0.17, s.e. = 0.04, *p* < 0.001; table 3). More importantly, people with stronger conservative leanings evaluated this coverage as more biased when they received it from CNN rather than Fox News (*β* = 0.15, s.e. = 0.04, *p* < 0.001; table 3 and figure 2*a*). Direction and statistical significance of results remain virtually unchanged with and without controls for prior media bias and attitudes towards the news content (see electronic supplementary material), supporting H1. However, figure 2*b* shows that the same pattern did not emerge in the case of neutral coverage of positive police conduct (i.e. police officer saves the life of a civilian) (*β* = 0.05, s.e. = 0.06, *p* = 0.129; table 3), corroborating the idea that news content (and how controversial it is) is relevant in triggering the HMP effect [8,9].

Furthermore, results suggest that HMP had no impact on news sharing intentions on social media, failing to support H2 (table 4 and figure 2*c*,*d*): neutral coverage from a source opposite to one's political leaning did not lead people to report different news sharing intentions either for news reporting on negative (*β* = −0.04, s.e. = 0.05, *p* = 0.417; table 4 and figure 2*c*) or positive (*β* = 0.01, s.e. = 0.05, *p* = 0.817; table 4 and figure 2*d*) police conduct. This is true regardless of the controls applied (see electronic supplementary material). In the electronic supplementary material, we show that similar patterns can be observed also for news sharing intentions in conversations (see section C.3). Finally, employing party identification instead of the conservative scale as an alternative measure of party leaning yields the same results both as regards news bias and news sharing intentions (see section C.2 in the electronic supplementary material).

## 5. Study 2: COVID-19 norms

### 5.1. Material and methods

#### 5.1.1. Experimental design

As in Study 1, subjects participated in a screening survey two to three weeks before the experiment. In the screening survey, we collected information on respondents' political orientation, beliefs on media bias, trust, attitudes concerning the legal system and demographics. The steps of the experiment were always the same: subjects were randomly assigned to a treatment, read the news from a mainstream outlet, and then filled out a questionnaire measuring news bias, sharing intentions and so on. In this 2 × 2 between-subjects design, we manipulated news source (Fox News versus CNN) and news content (people complying with versus protesting against COVID-19 norms), table 5.

**Table 5.** Between-subjects design of Study 2.

		news source	
		Fox News	CNN
news content	fellow citizens protesting social distancing orders	Fox News, fellow citizens protesting social distancing orders	CNN, fellow citizens protesting social distancing orders
	fellow citizens complying with social distancing orders	Fox News, fellow citizens complying with social distancing orders	CNN, fellow citizens complying with social distancing orders

**Figure 3.**
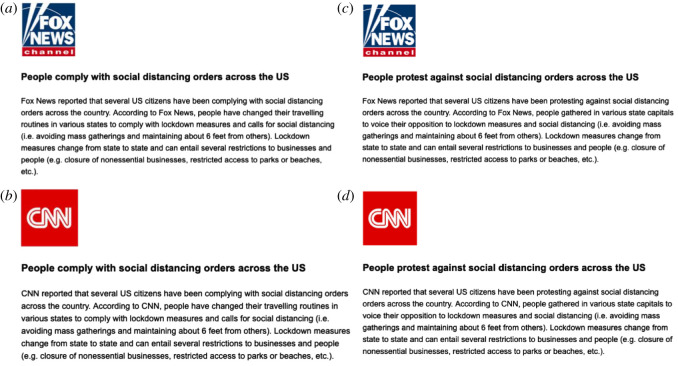
Treatments in Study 2 ‘COVID-19 norms’. (*a*) Fox News, people comply with COVID-19 norms; (*b*) CNN, people comply with COVID-19 norms; (*c*) Fox News, people protest against COVID-19 norms; (*d*) CNN, people protest against COVID-19 norms.

The news showed to participants followed the same criteria presented in Study 1 (short, neutral coverage of a real news item with a brief headline—about 600 characters max), figure 3. We provided the link to the original article upon request to all interested participants.

#### 5.1.2. Participants and procedures

Data were collected between 16 June and 12 July 2020. The questionnaire was prepared in Qualtrics and ran on AMT. We applied the same criteria for data collection described in Study 1 (i.e. recruitment of MTurkers with more than 98% of previous assignments approved and participation in at least 500 past assignments, only US participants, preventing re-takes). Only MTurkers who took part in the screening survey could participate in the experiment. The sample size was determined on the basis of previous studies and standards in the field [6,8,9,33,34].

The screening survey was completed by 995 participants who were then invited to the experiment; 821 MTurkers returned for the experiment (participation rate = 82.5%). Participants who did not answer questions measuring our dependent variables were excluded from the analyses, giving us a total sample size of 819 subjects for news bias and 818 subjects for news sharing intentions. Table 6 provides descriptive statistics of our sample.

**Table 6.** Study 2 ‘COVID-19 norms’ Descriptive statistics. CNN and Fox bias, as well as political leaning (i.e. conservative scale), COVID-19 attitudes, US division and demographics were measured in the screening survey.

variables	*M*	*s.d.*	*min*	*max*	*N*
news bias	1.56	0.86	0	4	819
news sharing intentions on social media	1.28	0.94	0	3	818
conservative scale	3.65	1.79	1	7	819
education					
high school diploma or less	0.08	0.27	0	1	819
some college	0.16	0.37	0	1	819
associate degree (2 years) or spec. technical training	0.10	0.30	0	1	819
bachelor's degree	0.45	0.50	0	1	819
some graduate training	0.03	0.16	0	1	819
graduate/professional degree or higher	0.18	0.38	0	1	819
ethnicity					
white	0.73	0.45	0	1	819
black	0.14	0.35	0	1	819
hispanic or latino/latina	0.03	0.18	0	1	819
asian	0.08	0.27	0	1	819
other	0.02	0.15	0	1	819
gender					
male	0.47	0.50	0	1	819
female	0.53	0.50	0	1	819
other	0.00	0.05	0	1	819
age	42.11	13.09	18	79	819
COVID-19: normative beliefs on social distancing	3.47	1.21	1	5	819
COVID-19: perceived compliance with social distancing	41.93	26.17	0	100	819
Fox bias	2.62	1.23	0	4	819
CNN bias	2.01	1.23	0	4	819
US Division					
New England	0.04	0.21	0	1	819
Mid-Atlantic	0.15	0.36	0	1	819
East North Central	0.15	0.36	0	1	819
West North Central	0.07	0.25	0	1	819
South Atlantic	0.20	0.40	0	1	819
East South Central	0.06	0.24	0	1	819
West South Central	0.09	0.29	0	1	819
Mountain	0.08	0.27	0	1	819
Pacific	0.15	0.35	0	1	819
Puerto Rico and other US territories	0.02	0.13	0	1	819

#### 5.1.3. Measures

We employed the same measures described in Study 1 for news bias (*α* = 0 .94), prior bias towards media outlet (Fox News bias *α* = 0 .98; CNN bias *α* = 0 .98), news sharing intentions in social media and in conversation, political leaning (i.e. conservative scale) and party identity. The conservative scale was centred around the mean in the regression models.

*COVID-19 attitudes.* Given the different news content, we used the following measures to control for prior attitudes towards COVID-19 norms, based on prior research on social norms and COVID-19 [37–42]: ‘Normative beliefs on social distancing' and ‘Perceived compliance with social distancing'. Normative beliefs on social distancing were measured using a 5-point scale (1 = strongly disagree, 5 = strongly agree) and it relied on the question: ‘People in the USA who have no symptoms of a Coronavirus infection should stay at home and avoid social contact to prevent the spread of the virus'. ‘Perceived compliance with social distancing' was based on the question ‘Given the current situation, out of 100 people, how many do you think stay at home and avoid social contact to prevent the spread of the virus if they have no symptoms of a Coronavirus infection (e.g. nose cold, sore throat, cough, fever)?', values range between 0 and 100.

## 6. Results and discussion

**Figure 4.**
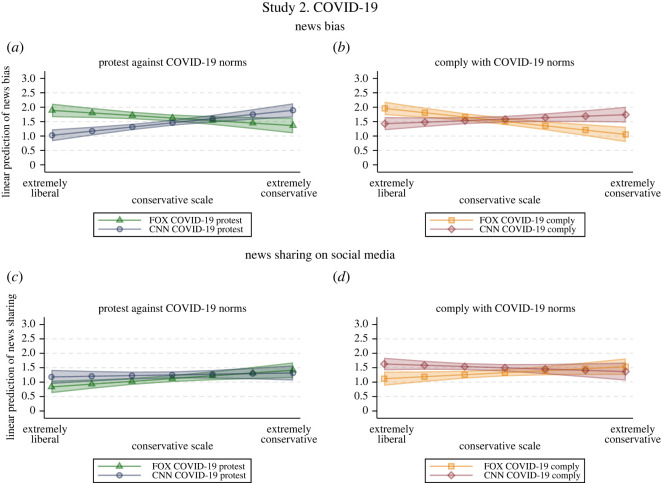
Study 2 ‘COVID-19 norms’—Linear prediction of news bias and news sharing intentions by political leaning and treatment. Linear prediction of perceived bias of news coverage (*N* = 819, *R^2^* = 0.20; (*a*,*b*)) and news sharing intentions on social media (*N* = 818, *R^2^* = 0.26; (*c*,*d*)) by political leaning and treatment with 95% CIs based on OLS regression with robust standard errors and controls for demographics, US state, prior media bias and attitudes towards the news content.

Using the same analytical strategy employed in Study 1, we estimate the impact of the interaction between political leaning and treatment condition on news bias and news sharing intentions. In tables 7 and 8, we report the difference between the slopes of political leaning across treatment conditions with the same content (e.g. Comply with COVID-19 norms) but different news source (i.e. Fox News versus CNN) [36]. Figure 4 shows the linear prediction of perceived bias of news coverage (*N* = 819, *R^2^* = 0 .20; (*a*,*b*)) and news sharing intentions on social media (*N* = 818, *R^2^* =0 .26; (*c*,*d*)) by treatment and political leaning with controls for prior media bias and prior attitudes concerning COVID-19, as well as US State, and demographics. See the electronic supplementary material for detailed regression results and robustness checks.

**Table 7.** Study 2 ‘COVID-19 norms’—Effect of conservative leaning on news bias within and between treatments. Linear effects within and between treatments are computed using average marginal effects in Stata 16.1 employing the *margins* and *mlincom* commands. Robust standard errors in parentheses.

DV: news bias	linear effect within treatments (first difference)		linear effect between treatments (second difference)
conservative in Fox COVID protest	−0.088* (0.038)	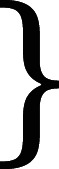	0.232*** (0.046)
conservative in CNN COVID protest	0.144*** (0.034)		
conservative in Fox COVID comply	−0.150*** (0.036)	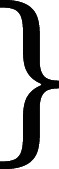	0.203*** (0.050)
conservative in CNN COVID comply	0.052 (0.038)		

*Note.* *p ≤ 0.05, **p ≤ 0.01, ***p ≤ 0.001 (for two-sided tests).

**Table 8.** Study 2 ‘COVID-19 norms’—Effect of conservative leaning on news sharing intentions on social media within and between treatments. Linear effects within and between treatments are computed using average marginal effects in Stata 16.1 employing the *margins* and *mlincom* commands. Robust standard errors in parentheses.

DV: news sharing	linear effect within treatments (first difference)		linear effect between treatments (second difference)
conservative in Fox COVID protest	0.096** (0.036)	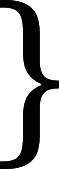	−0.073 (0.046)
conservative in CNN COVID protest	0.023 (0.038)		
conservative in Fox COVID comply	0.069 (0.040)	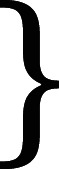	−0.113* (0.051)
conservative in CNN COVID comply	−0.044 (0.040)		

*p ≤ 0.05, **p ≤ 0.01, ***p ≤ 0.001 (for two-sided tests).

We can observe a clear HMP effect on news bias both for news reporting on people complying with and protesting against COVID-19 norms. Stronger conservatives perceived the same neutral coverage as more biased when the source was CNN instead of Fox News, while the opposite was true for stronger liberals (figure 4*a*,*b*): a one-unit increase on the conservative scale yields a rise in perceived news bias of 0.23 points when the source is CNN rather than Fox for identical news concerning protests against COVID-19 norms (*β* = 0.23, s.e. = 0.05, *p* < 0.001; table 7), and of 0.20 points for identical news concerning compliance with COVID-19 norms (*β* = 0.20, s.e. = 0.05, *p* < 0.001; table 7). That is, when faced with identical news coverage concerning COVID-19, conservatives' and liberals' perception of news bias seems to heavily depend on whether the source of information was aligning with their own political view. This holds true with and without controls for prior media bias or attitudes towards COVID-19 (see electronic supplementary material), supporting H1.

As regards news sharing intentions, evidence only partially supports HMP theory: people with stronger conservative leanings reported a higher (marginally significant) willingness to share news from Fox News rather than CNN when the news concerns COVID-19 complying behaviours (*β* = 0.11, s.e. = 0.05, *p* < 0.05; table 8 and figure 4*d*). The same did not emerge when the news was about protests against COVID-19 norms (*β* =0.07, s.e. = 0.05, *p* = 0.110; table 8 and figure 4*c*). This evidence does not convincingly support H2, and it suggests that the scope of HMP could be more limited than what was theoretically argued. Models with and without controls for prior media bias or attitudes towards COVID-19 norms support the same conclusions (see the electronic supplementary material; see section C.3 for results on news sharing intentions in conversations), and employing party identification as an alternative measure of party leaning yields once again the same results both as regards news bias and news sharing intentions (see section C.2 in the electronic supplementary material).

## 7. General discussion

Our study provides support for the HMP effect on news bias but not on news sharing intentions. Our findings show that, in the US, partisans exposed to the same neutral coverage of news by outlets opposite to their political leaning perceived the news as more biased, even when accounting for their prior beliefs regarding the media outlet and news content. These results reinforce the idea that HMP is driven by political motivations rather than prior attitudes towards the news content or the media outlet.

We show that news reporting on important contemporary and debated issues in US society, such as negative police conduct (Study 1) and compliant or defiant behaviours concerning COVID-19 norms (Study 2), are more likely to be perceived as biased when the news source is not aligned with one's political view. This HMP effect might contribute to further polarization in US society, eroding trust towards media outlets and information providers. However, in line with theory, our evidence indicates that not all news content is relevant in triggering HMP: when respondents are exposed to less controversial news (i.e. positive police conduct, Study 1), perceived news bias is similar across political leanings.

Furthermore, our study points out that news sharing intentions are not significantly affected by news source. Indeed, in both studies respondents did not show a distinct lower or higher propensity for sharing a certain news item depending on the news source. Only in the case of news reporting on complying behaviours regarding COVID-19 norms we observed some weak support for an HMP effect on news sharing intentions. Such a discrepancy between what people believe about the credibility of a news and what they are willing to share is consistent with research on misinformation and fake news, which indicates that (politically motivated) agreement with news content is one of the most relevant factors in shaping how people spread information [43–46]. Overall, our analysis corroborates the robustness of the HMP effect on news bias, while pointing out that the scope of HMP on broader attitudes and behavioural intentions may be more limited than what was theoretically postulated.

Nevertheless, this study does not measure sharing behaviour (e.g. on Twitter or Facebook) nor give respondents the opportunity to share the news they have been exposed to. While our measure of sharing intentions has been shown to reasonably approximate actual sharing behaviour on social media [35], we could not test if exposure to neutral coverage of news by mainstream outlets opposite to one's political leaning was associated with a change in respondents' sharing activity for that news. By linking survey responses with participants' Twitter or Facebook accounts, future research could further explore the impact of HMP on sharing behaviour as well as tie formation, social media use and media diets. For instance, as various studies have found that individuals tend to acquire new information consistent with their initial attitude when they feel conflicted [47–49], it could be explored whether HMP encourages a switch to other social platforms (e.g. Telegram) or media diets that are more in line with individuals' initial attitudes. This could be an interesting avenue for future research as different countries, such as Germany, are considering the possibility to impose stricter regulations or banish some social platforms to tackle extremism and misinformation [50–52].

Also, along the lines of recent work from Guess and colleagues [53,54], it would be interesting to explore, in a longitudinal field experiment, if an increase in the strength of exposure (e.g. in terms of number of news items) to neutral coverage from sources perceived of opposite political leaning produces attitudinal (e.g. a lower bias towards the outlet) or behavioural changes (e.g. more frequent sharing of news from other sources, more diverse or attitude-consistent media diets) that ‘stick' with the participants, even after the end of the intervention.

## Data Availability

The dataset analysed during the current study is available on the Open Science Framework website at the following link: https://osf.io/5rzvx/.

## References

[1] Hanitzsch T, Van Dalen A, Steindl N. 2018 Caught in the nexus: a comparative and longitudinal analysis of public trust in the press. Int. J. Press/Politics **23**, 3-23. (10.1177/1940161217740695)

[2] Lee T-T. 2010 Why they don't trust the media: an examination of factors predicting trust. Am. Behav. Scientist **54**, 8-21. (10.1177/0002764210376308)

[3] Mitchell A, Gottfried J, Kiley J, Matsa K. 2014 Political polarization & media habits. Washington, DC: Pew Research Center, Journalism and Media.

[4] Pennycook G, Rand DG. 2019 Fighting misinformation on social media using crowdsourced judgments of news source quality. Proc. Natl Acad. Sci. USA **116**, 2521-2526. (10.1073/pnas.1806781116) 30692252 PMC6377495

[5] Baum MA, Gussin P. 2007 In the eye of the beholder: how information shortcuts shape individual perceptions of bias in the media. Q. J. Political Sci. **3**, 1-31. (10.1561/100.00007010)

[6] Feldman L. 2011 Partisan differences in opinionated news perceptions: a test of the hostile media effect. Political Behav. **33**, 407-432. (10.1007/s11109-010-9139-4)

[7] Gunther AC, Schmitt K. 2004 Mapping boundaries of the hostile media effect. J. Commun. **54**, 55-70. (10.1111/j.1460-2466.2004.tb02613.x)

[8] Feldman L. 2012 The hostile media effect. In The Oxford handbook of political communication (eds K Kenski, K Hall Jamieson), pp. 549-564. New York NY: Oxford University Press.

[9] Perloff RM. 2015 A three-decade retrospective on the hostile media effect. Mass Commun. Soc. **18**, 701-729. (10.1080/15205436.2015.1051234)

[10] Arpan LM, Raney AA. 2003 An experimental investigation of news source and the hostile media effect. Journalism Mass Commun. Q. **80**, 265-281. (10.1177/107769900308000203)

[11] Mills CE. 2017 Framing Ferguson: Fox News and the construction of US racism. Race Class **58**, 39-56. (10.1177/0306396816685030)

[12] Mourtgos SM, Adams IT. 2020 Assessing public perceptions of police use-of-force: legal reasonableness and community standards. Justice Q. **37**, 869-899. (10.1080/07418825.2019.1679864)

[13] Thomas MD, Reeves AN, Jewell NP, Michaels EK, Allen AM. 2021 US law enforcement policy predictors of race-specific police fatalities during 2015–16. PLoS ONE **16**, e0252749. (10.1371/journal.pone.0252749) 34161363 PMC8221500

[14] Benan M. 2021 *Americans’ confidence in major U.S. institutions dips*. Gallup Poll Social Series. See https://news.gallup.com/poll/352316/americans-confidence-major-institutions-dips.aspx.

[15] Pew Research Center. 2020 Majority of public favors giving civilians the power to sue police officers for misconduct. Pew Research Center. See https://www.pewresearch.org/politics/2020/07/09/majority-of-public-favors-giving-civilians-the-power-to-sue-police-officers-for-misconduct/.

[16] Jones MJ. 2015 In U.S., confidence in police lowest in 22 years. Gallup Poll Social Series. See https://news.gallup.com/poll/183704/confidence-police-lowest-years.aspx.

[17] Brodeur A, Gray D, Islam A, Bhuiyan S. 2021 A literature review of the economics of COVID-19. J. Econ. Surv. **35**, 1007-1044. (10.1111/joes.12423) 34230772 PMC8250825

[18] Green J, Edgerton J, Naftel D, Shoub K, Cranmer SJ. 2020 Elusive consensus: polarization in elite communication on the COVID-19 pandemic. Sci. Adv. **6**, eabc2717. (10.1126/sciadv.abc2717) 32923600 PMC7455486

[19] Tappin BM, Pennycook G, Rand DG. 2020 Thinking clearly about causal inferences of politically motivated reasoning: why paradigmatic study designs often undermine causal inference. Curr. Opin. Behav. Sci. **34**, 81-87. (10.1016/j.cobeha.2020.01.003)

[20] Van Bavel JJ, Pereira A. 2018 The partisan brain: an identity-based model of political belief. Trends Cogn. Sci. **22**, 213-224. (10.1016/j.tics.2018.01.004) 29475636

[21] Peterson E, Kagalwala A. 2021 When unfamiliarity breeds contempt: how partisan selective exposure sustains oppositional media hostility. Am. Political Sci. Rev. **115**, 585-598. (10.1017/S0003055420001124)

[22] Gerber A, Green D. 1999 Misperceptions about perceptual bias. Annu. Rev. Political Sci. **2**, 189-210. (10.1146/annurev.polisci.2.1.189)

[23] Levendusky MS. 2013 Why do partisan media polarize viewers? Am. J. Political Sci. **57**, 611-623. (10.1111/ajps.12008)

[24] Yale RN, Jensen JD, Carcioppolo N, Sun Y, Liu M. 2015 Examining first-and second-order factor structures for news credibility. Commun. Methods Measures **9**, 152-169. (10.1080/19312458.2015.1061652)

[25] Buhrmester M, Kwang T, Gosling SD. 2016 Amazon's Mechanical Turk: A new source of inexpensive, yet high-quality data? Perspect. Psychol. Sci. **6**, 3-5. (10.1177/1745691610393980) 26162106

[26] Casler K, Bickel L, Hackett E. 2013 Separate but equal? A comparison of participants and data gathered via Amazon's MTurk, social media, and face-to-face behavioral testing. Comput. Hum. Behav. **29**, 2156-2160. (10.1016/j.chb.2013.05.009)

[27] Hauser D, Paolacci G, Chandler JJ. 2018 Common concerns with MTurk as a participant pool: evidence and solutions. PsyArXiv. (10.31234/osf.io/uq45c)

[28] Aruguete MS, Huynh H, Browne BL, Jurs B, Flint E, McCutcheon LE. 2019 How serious is the ‘carelessness’ problem on Mechanical Turk? Int. J. Soc. Res. Methodol. **22**, 441-449. (10.1080/13645579.2018.1563966)

[29] Chmielewski M, Kucker SC. 2020 An MTurk crisis? Shifts in data quality and the impact on study results. Social Psychol. Pers. Sci. **11**, 464-473. (10.1177/1948550619875149)

[30] Peer E, Vosgerau J, Acquisti A. 2014 Reputation as a sufficient condition for data quality on Amazon Mechanical Turk. Behav. Res. Methods **46**, 1023-1031. (10.3758/s13428-013-0434-y) 24356996

[31] Robinson J, Rosenzweig C, Moss AJ, Litman L. 2019 Tapped out or barely tapped? Recommendations for how to harness the vast and largely unused potential of the Mechanical Turk participant pool. PLoS ONE **14**, e0226394. (10.1371/journal.pone.0226394) 31841534 PMC6913990

[32] Cheung JH, Burns DK, Sinclair RR, Sliter M. 2017 Amazon Mechanical Turk in organizational psychology: an evaluation and practical recommendations. J. Bus. Psychol. **32**, 347-361. (10.1007/s10869-016-9458-5)

[33] Lee TK, Kim Y, Coe K. 2018 When social media become hostile media: an experimental examination of news sharing, partisanship, and follower count. Mass Commun. Soc. **21**, 450-472. (10.1080/15205436.2018.1429635)

[34] Clavio G, Vooris R. 2018 ESPN and the hostile media effect. Commun. Sport. **6**, 728-744. (10.1177/2167479517739835)

[35] Mosleh M, Pennycook G, Rand DG. 2020 Self-reported willingness to share political news articles in online surveys correlates with actual sharing on Twitter. PLoS ONE **15**, e0228882. (10.1371/journal.pone.0228882) 32040539 PMC7010247

[36] Mize TD. 2019 Best practices for estimating, interpreting, and presenting nonlinear interaction effects. Sociol. Sci. **6**, 81-117. (10.15195/v6.a4)

[37] Bicchieri C. 2016 Norms in the wild: How to diagnose, measure, and change social norms. Oxford, UK: Oxford University Press.

[38] Bicchieri C, Lindemans JW, Jiang T. 2014 A structured approach to a diagnostic of collective practices. Front. Psychol. **5**, 1418. (10.3389/fpsyg.2014.01418) 25538666 PMC4257103

[39] de Bruin WB, Bennett D. 2020 Relationships between initial COVID-19 risk perceptions and protective health behaviors: a national survey. Am. J. Prev. Med. **59**, 157-167. (10.1016/j.amepre.2020.05.001) 32576418 PMC7242956

[40] de Wit JR, Lisciandra C. 2020 Measuring norms using social survey data. Econ. Phil. **37**, 1-34.

[41] Van Bavel JJ et al. 2020 Using social and behavioural science to support COVID-19 pandemic response. Nat. Hum. Behav. **4**, 460-471. (10.1038/s41562-020-0884-z) 32355299

[42] Lo Iacono S, Przepiorka W, Buskens V, Corten R, van de Rijt A. 2021 COVID-19 vulnerability and perceived norm violations predict loss of social trust: a pre-post study. Soc. Sci. Med. **291**, 114513. (10.1016/j.socscimed.2021.114513) 34717284 PMC8553419

[43] Jerit J, Zhao Y. 2020 Political misinformation. Annu. Rev. Political Sci. **23**, 77-94. (10.1146/annurev-polisci-050718-032814)

[44] Mosleh M, Pennycook G, Arechar AA, Rand DG. 2021 Cognitive reflection correlates with behavior on Twitter. Nat. Commun. **12**, 1-10. (10.1038/s41467-020-20043-0) 33568667 PMC7875970

[45] Pennycook G, Rand DG. 2019 Lazy, not biased: susceptibility to partisan fake news is better explained by lack of reasoning than by motivated reasoning. Cognition **188**, 39-50. (10.1016/j.cognition.2018.06.011) 29935897

[46] Pennycook G, Rand DG. 2021 The psychology of fake news. Trends Cogn. Sci. **25**, 388-402. (10.1016/j.tics.2021.02.007) 33736957

[47] Itzchakov G, Van Harreveld F. 2018 Feeling torn and fearing rue: attitude ambivalence and anticipated regret as antecedents of biased information seeking. J. Exp. Soc. Psychol. **75**, 19-26. (10.1016/j.jesp.2017.11.003)

[48] Jonas K, Diehl M, Brömer P. 1997 Effects of attitudinal ambivalence on information processing and attitude-intention consistency. J. Exp. Soc. Psychol. **33**, 190-210. (10.1006/jesp.1996.1317)

[49] Maio GR, Bell DW, Esses VM. 1996 Ambivalence and persuasion: the processing of messages about immigrant groups. J. Exp. Soc. Psychol. **32**, 513-536. (10.1006/jesp.1996.0023) 8979932

[50] Reuters. Politician says Germany should ban Telegram unless it tackles extremist content. 2021 See https://wwwreuterscom/world/europe/politician-says-germany-should-ban-telegram-unless-it-tackles-extremist-content-2021-12-14/.

[51] BBC. 2019 Websites to be fined over ‘online harms’ under new proposals. See https://wwwbbccom/news/technology-47826946.

[52] BBC. 2020 Social media: How might it be regulated? See https://wwwbbccom/news/technology-54901083.

[53] Guess AM. 2021 (Almost) everything in moderation: new evidence on Americans’ online media diets. Am. J. Political Sci. **65**, 1007-1022. (10.1111/ajps.12589)

[54] Guess AM, Barberá P, Munzert S, Yang J. 2021 The consequences of online partisan media. Proc. Natl Acad. Sci. USA **118**, e2013464118. (10.1073/pnas.2013464118) PMC8040813 33782116

